# Supplementary material for: Microencapsulation of Anthocyanins from Zea mays and Solanum tuberosum: Impacts on Antioxidant, Antimicrobial, and Cytotoxic Activities
Source: Nutrients. 2024 Nov 27;16(23):4078. doi: 10.3390/nu16234078 (PMC11643658; doi:10.3390/nu16234078)
Supplement: Supplementary file 1 [file nutrients-16-04078-s001.zip › nutrients-3306536-supplementary.pdf]

A

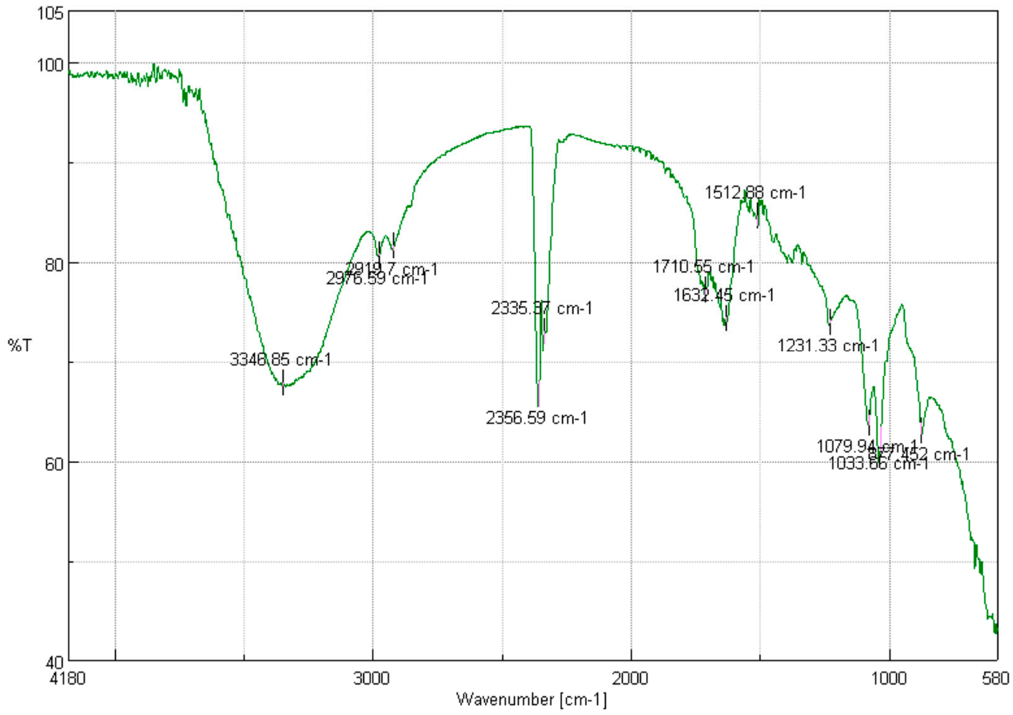

B

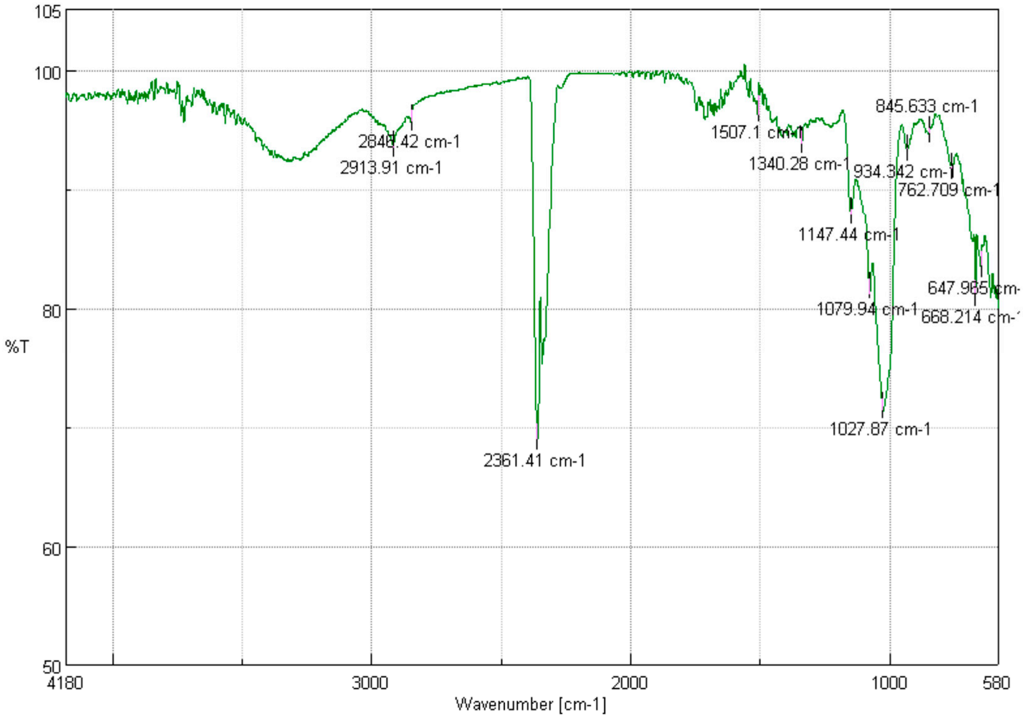

Figure S1: FTIR Spectral Curves of Anthocyanins from *Solanum tuberosum* L. A) FTIR spectrum of non-encapsulated anthocyanins of *S. tuberosum* L. The spectrum displays the characteristic absorption bands corresponding to the functional groups present in the anthocyanins, indicating the molecular structure of the non-encapsulated form. B) FTIR spectrum of microencapsulated anthocyanins from *S. tuberosum* L. The encapsulation process alters the spectral profile, suggesting changes in molecular interactions or conformations due to the encapsulation matrix.

A

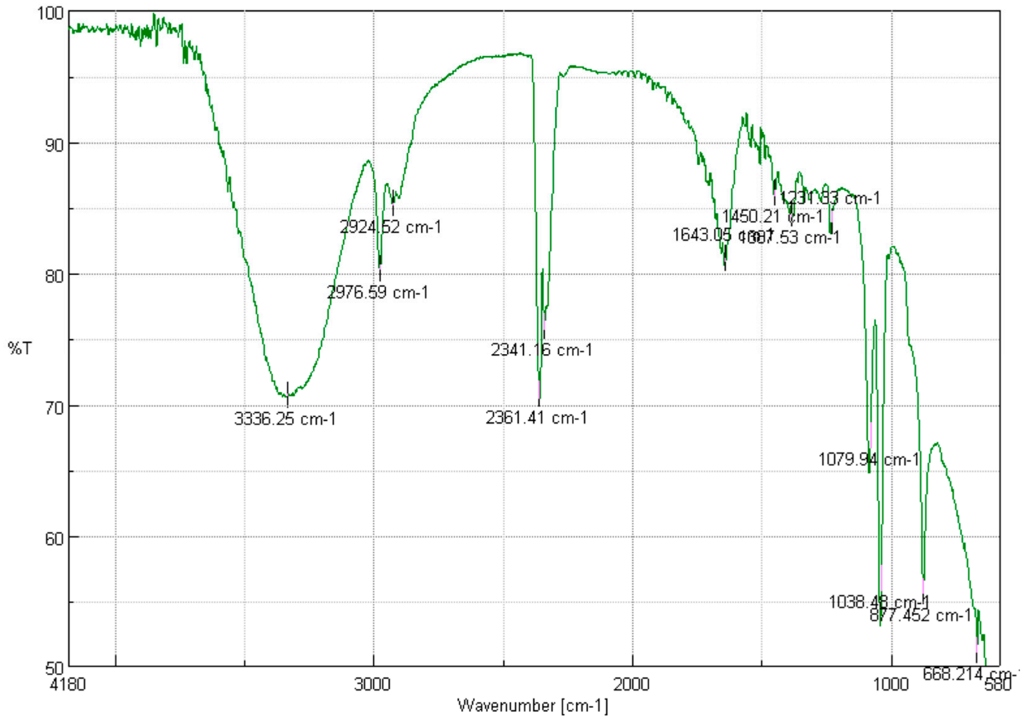

B

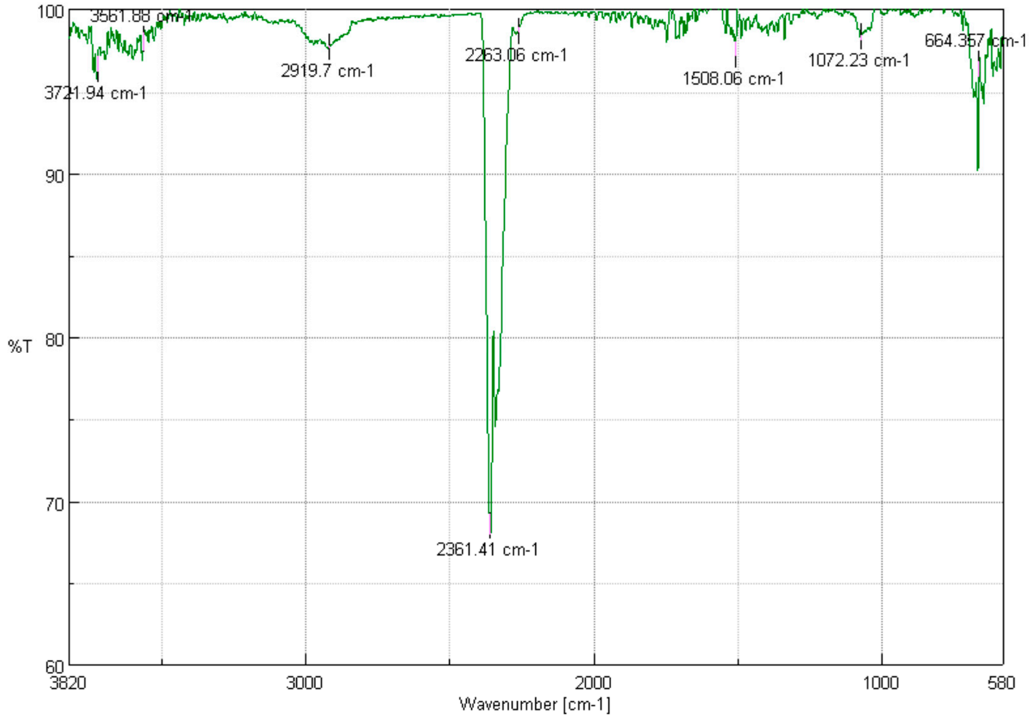

Figure S2: FTIR Spectral Curves of Anthocyanins from *Zea mays* L. A) FTIR spectrum of non-encapsulated anthocyanins of *Z. mays* L. The spectrum displays the characteristic absorption bands corresponding to the functional groups present in the anthocyanins, indicating the molecular structure of the non-encapsulated form. B) FTIR spectrum of microencapsulated anthocyanins from *Z. mays* L. The encapsulation process alters the spectral profile, suggesting changes in molecular interactions or conformations due to the encapsulation matrix.

Table S1 .Antioxidant activity of the microencapsulated anthocyanins from *Zea mays* L. and *Solanum tuberosum* L

**Concentration (µg/mL)**	**DPPH %**	
	S. tuberosum	***Z. mays***
10	15	12
50	28	20
100	38	30
200	48	37
300	55	43
400	61	48
500	65	52

DPPH: 2,2-diphenyl-1-picrylhydrazyl

Table S2. Apoptosis index (AI) of HeLa cells treated with microencapsulated anthocyanins of *Zea mays* L and *S. tuberosum* L

Condition	*AI (mean ± SD)*
Control	2.32 ± 1.02
Zea mays L.	2.47 ± 0.95
S. tuberosum L.	2.60 ± 0.90
